# Arabinogalactan Proteins and the Extracellular Matrix of Charophytes: A Sticky Business

**DOI:** 10.3389/fpls.2019.00447

**Published:** 2019-04-12

**Authors:** Kattia Palacio-López, Berke Tinaz, Andreas Holzinger, David S. Domozych

**Affiliations:** ^1^Department of Biology, Skidmore College, Saratoga Springs, NY, United States; ^2^Department of Botany, University of Innsbruck, Innsbruck, Austria

**Keywords:** adhesion, arabinogalactan protein, monoclonal antibody, charophyte, cell wall, extracellular matrix

## Abstract

Charophytes represent the group of green algae whose ancestors invaded land and ultimately gave rise to land plants 450 million years ago. While Zygnematophyceae are believed to be the direct sister lineage to embryophytes, different members of this group (*Penium*, *Spirogyra*, *Zygnema*) and the advanced thallus forming *Coleochaete* as well as the sarcinoid basal streptophyte *Chlorokybus* were investigated concerning their vegetative extracellular matrix (ECM) properties. Many taxa exhibit adhesion phenomena that are critical for affixing to a substrate or keeping cells together in a thallus, however, there is a great variety in possible reactions to e.g., wounding. In this study an analysis of adhesion mechanisms revealed that arabinogalactan proteins (AGPs) are most likely key adhesion molecules. Through use of monoclonal antibodies (JIM13) or the Yariv reagent, AGPs were located in cell surface sheaths and cell walls that were parts of the adhesion focal zones on substrates including wound induced rhizoid formation. JIM5, detecting highly methyl-esterfied homoglacturonan and JIM8, an antibody detecting AGP glycan and LM6 detecting arabinans were also tested and a colocalization was found in several examples (e.g., *Zygnema*) suggesting an interplay between these components. AGPs have been described in this study to perform both, cell to cell adhesion in algae forming thalli and cell to surface adhesion in the filamentous forms. These findings enable a broader evolutionary understanding of the function of AGPs in charophyte green algae.

## Introduction

The extracellular matrix (ECM) of plants is composed of highly complex domains of biopolymers, enzymes, ions and water that integrate into diverse architectural designs and form an effective interface between the cell surface and external environment. The matrix also dynamically modulates throughout the life of the cell in response to both internal genetic programs and external signals/stresses. The plant ECM may be dissected into two domains, the cell wall, and “components deposited onto the cell wall surface” and/or secreted beyond the wall. The cell wall can be described as a fiber reinforced hydrogel (Ali and Traas, 2016) consisting primarily of polysaccharides and proteoglycans. The main load-bearing component of the wall is crystalline cellulose organized in approximately 3 nm-wide microfibrils that are cross-linked by, and embedded in, a matrix of neutral polysaccharides (hemicelluloses), acidic polysaccharides (pectins) and proteoglycans (extensins, AGPs) (Braidwood et al., 2014; Cosgrove, 2014). The cell wall most often contains various ions that contribute to its structural integrity (Shomer et al., 2003) and in secondary cell walls, lignins generally impregnate the wall infrastructure (Marriott et al., 2016; Özparpucu et al., 2017). External to the wall exists a highly diverse assortment of biochemical components including lipid cuticles (Yeats and Rose, 2013), post-germination seed gels (Francoz et al., 2015) and root mucilages (Walker et al., 2003). The functions of the ECM are of paramount importance to the plant as it provides physical and chemical protection, a communication conduit between cells and their biotic/abiotic surroundings, a means to hold water (i.e., desiccation avoidance), a turgor-balancing control for cell expansion and a highway for translocation of water and minerals (Lionetti and Métraux, 2014; Bidhendi and Geitmann, 2016; Chebli and Geitmann, 2017). It is therefore not surprising that plant cells expend large amounts of photosynthetic carbon and devote up to 30% of their genetic machinery to the synthesis of the ECM.

Many components of the ECM found in embryophytes or “land plants” are also found in the charophyte green algae (or basal Streptophytes), i.e., the group of extant green algae whose ancestors gave rise to land plants between 450 and 500 million years ago (Becker and Marin, 2009; Leliaert et al., 2012; Delwiche and Cooper, 2015). Recent biochemical and immuno-binding studies have demonstrated the presence of cellulose, xyloglucans, ß,1-3/1-4 glucans, pectins, extensin, AGP and even lignin-like components in the cell walls of charophytes (Sørensen et al., 2011; Domozych et al., 2012; De Vries et al., 2017; Herburger et al., 2018). Additionally, many charophytes secrete large amounts of complex extracellular polysaccharides (EPS) beyond their cell walls (Kiemle et al., 2007) that are involved in photomotility, substrate attachment, anti-desiccation and biofilm formation (Boney, 1981; Oertel et al., 2004; Domozych et al., 2005; Domozych and Domozych, 2008). However, we are only just beginning to decipher the detailed structure, location and function of the specific components of the charophyte ECM. Further studies would yield valuable insight into the molecular and cellular adaptations that may have occurred during the emergence of green plants onto land and the subsequent proliferation of plants into diverse terrestrial habitats. Furthermore, many charophytes are very attractive model organisms for ECM studies as their small size, rapid growth rates and ease in laboratory culture manipulation provide superb systems for experimental analyses at all levels ranging from transcriptome analysis (Rippin et al., 2017; Van De Poel et al., 2016) to cell wall composition (Brosch-Salomon et al., 1998; Lütz-Meindl and Brosch-Salomon, 2000; Eder and Lütz-Meindl, 2008; Eder et al., 2008; Proseus and Boyer, 2008; Domozych, 2014; Domozych et al., 2014, 2016; Boyer, 2016; Lütz-Meindl, 2016) and ecology.

Adhesion is a phenomenon that is essential for most living organisms. This process is central to a diverse array of biological processes ranging from the establishment of multicellularity (Grosberg and Strathmann, 2007; Olson and Nedelcu, 2016) to the formation of a sessile habit (Morrison et al., 2009) and to the development of biofilms (Jain et al., 2007). While some adhesion phenomena require specialized cells for sustaining close contact with a substrate (e.g., rhizoids), most are directly associated with the structural architecture and dynamic modulation of the ECM. Cell adhesion molecules or CAMs are the specific components of the ECM, typically proteins or polysaccharides that are integral to the adhesion process. Their unique biochemical and structural properties are often precisely designed for interaction with a particular substrate. In plants, CAMs are components of the ECM that work in close concert with a substrate, cell wall polymers, the plasma membrane and the cortical cytoskeleton/cytoplasm (Baluška et al., 2003; Huang et al., 2016; Langhans et al., 2017). Adhesion phenomena are also commonly found in charophytes and are perhaps best exemplified by the cell/thallus establishing close contact with, and attaching to, a stationary substrate. This is a critical step for transition from the planktonic to sessile state, maintaining an optimal position for light acquisition for photosynthesis and/or living in a complex biofilm community in a frequently changing habitat like an ephemeral wetland, i.e., common homes of both modern day and ancient charophytes.

Recent studies have demonstrated that AGPs are integral to many adhesion-based mechanisms (Groot et al., 2003; Bowling and Vaughn, 2008; Huang et al., 2016). AGPs represent a diverse group of highly glycosylated proteins that are found in the cell wall, the apoplastic space and/or the medium surrounding plant cell. They are widely distributed throughout the major taxa of land plants (Šamaj et al., 1999b; Bartels and Classen, 2017; Bartels et al., 2017; Johnson et al., 2017b; Ma et al., 2017). AGPs belong to a large group of hydroxyproline (hyp)-containing proteins and possess a protein backbone consisting of repeating subunits that contain hyp/proline (pro), alanine (ala), and serine (ser)/threonine (thr) (Johnson et al., 2003, 2017a,b; Lamport et al., 2014, 2018; Herve et al., 2016; Bartels et al., 2017). The hyp is often *O*-glycosylated with ß 1–3 or ß1–6 or ß 3–6 galactan chains to which are attached other sugars including glucuronic acid. The carbohydrate component of AGPs is remarkably diverse amongst different taxa and often constitutes up to 99% of the whole molecule. Recent work has also shown that AGPs may be structurally and/or functionally associated with other polymers leading to the supposition that they may be part of large polymer domains of the cell wall that include pectins and xylans (Tan et al., 2012, 2013). The functions of AGPs are many and diverse. In addition to their roles in adhesion (Šamaj et al., 1999a,c; Huang et al., 2016; Langhans et al., 2017), they also contribute to cell expansion (Yang et al., 2007) pollen tube dynamics during fertilization (Nguema-Ona et al., 2012; Pereira et al., 2015, 2016; Lamport et al., 2018) cell expansion (van Hengel and Roberts, 2002; Seifert and Roberts, 2007), calcium dynamics during development (Majewska-Sawka and Nothnagel, 2000; Oxley and Bacic, 2000; Lamport and Varnai, 2012; Lamport et al., 2018), salt tolerance (Olmos et al., 2017), and many others (Seifert and Roberts, 2007; Ellis et al., 2010).

AGP-like proteoglycans have been demonstrated in charophytes (Sørensen et al., 2011; Ruiz-May et al., 2018) including desmids (Lütz-Meindl and Brosch-Salomon, 2000; Domozych et al., 2007; Eder et al., 2008) and *Chara* (Domozych et al., 2010, 2009) and have been implicated in various cellular events including adhesion. However, knowledge of their distribution amongst the various charophyte taxa and inclusive morphotypes is limited and their roles in adhesion are poorly resolved. In this study, we examined five taxa from three major groups of charophytes, the early divergent Chlorokybales and the late divergent Coleochaetales and Zygnematales that exhibit distinct adhesion phenomena. Employing a variety of labeling protocols and experimental techniques, we show that AGP-like macromolecules are involved in various adhesion phenomena.

## Materials and Methods

### General

Live cultures of algae were maintained at 20^o^C, 16 h light/8 h dark with 74 μmol photons m^-2^ s^-1^ of cool white fluorescent light in liquid cultures containing the following media: *Penium margaritaceum* Skidmore College Collection: SKD-8 (Woods Hole Medium with 5% soil extract; Domozych et al., 2017), *Chlorokybus atmophyticus* UTEX 2591 (Woods Hole Medium with 10% soil extract), *Zygnema* *circumcarinatum* SAG 2419 (Herburger et al., 2019, for this study grown in 3N BBM^1^), *Spirogyra* sp. Carolina Biological 152525 (3N BBM) and *Coleochaete orbicularis* UTEX 2651 (Woods Hole medium with 5% soil extract and 1% peat extract). Cells were harvested for labeling and experiments 10–14 days after subculturing, in the case of *Zygnema* also older cultures (3–6 months) were used for transmission electron microscopy. Cells were concentrated by centrifugation at 700–1,000 ×*g* for 1 min. Washing consisted of resuspending centrifuged pellets in fresh growth medium, shaking and recentrifuging. This step was repeated three times before labeling.

#### Wound Response of Rhizoids

*Spirogyra* sp. filaments were removed from culture and placed on the bottom of a sterile plastic petri dish. The filaments were chopped to small fragments with a sterile razor blade. Masses of chopped filaments were then added to a sterile petri dish containing 3N BBM with sterile 22 × 22 mm coverslips lining the bottom. The petri dishes were cultured as described above. Within 24 h rhizoids emerged from the wounded filaments and attached to the coverslips. The coverslips containing the rhizoids were used for labeling. A similar wounding protocol was employed for *Zygnema circumcarinatum* but no rhizoids or adhesion to coverslips were observed.

### Fluoresbrite Bead Labeling

The following protocols were employed in order to screen for adhesive ECM components. Cells/thalli that were either attached to a surface (e.g., glass coverslip, plastic petri dish) or planktonic were collected and washed with fresh growth medium in order to remove any pre-existing materials from the cell surface that might interfere with subsequent experiments. They were then incubated in a solution of 50 μL 0.5 μm Fluoresbrite beads (Polysciences, United States)/mL growth medium for 15 min with gently shaking. Cells/thalli or substrates with attached algae were washed 3× with fresh growth medium to remove excess beads. Cells or substrates were mounted on glass slides and viewed with wide field fluorescence labeling (WFLM) equipped with a FITC filter set. For *Spirogyra*, coverslips containing rhizoids were collected, washed in a stream of fresh growth medium and then incubated in the Fluoresbrite solution. Cells were also placed on plastic petri dishes (*Penium*, *Chlorokybus*) were allowed to settle onto the surface. They were then labeled with the Fluoresbrite beads or cells were gently removed with a fine stream of growth medium and the sites of attachment were incubated in drops of the beads. All samples were viewed with an inverted light microscopy (LM) equipped with a FITC filter set.

### ß-Glucosyl-Yariv Reagent Labeling and Growth Inhibition Analyses

Washed cells or coverslips were incubated for 60 min in 10 and 20 μM ß-glucosyl-Yariv (BioSupplies, United States), washed with growth medium and examined with LM. As a control, algae were labeled with β-galactosyl Yariv and imaged. This reagent does not bind to AGP. For inhibition experiments, cells or wound induced filaments were cultured in growth medium containing 20 μM ß-glucosyl-Yariv (or β-galactosyl Yariv as a control). Cells were collected 24, 36, and 96 h after treatment and incubated with Fluoresbrite beads or allowed to settle on the base of a plastic petri dish or glass coverslip to observe adhesion properties. For *Spirogyra* sp. rhizoid analysis, cut filaments were placed in petri dishes with coverslips as described above and cultured in the Yariv reagents. Examination of adhesion efficacy was then monitored by LM.

### Immunofluorescence Labeling

Harvested cells were washed three times with fresh growth medium and labeled for immunofluorescence as described in Domozych et al. (2014, 2017). The primary antibodies used were obtained from Plant Probes (Leeds, United Kingdom^2^) and included JIM5 (specificity: Homogalacturonan, HG, with low degree of esterification), JIM13 (sp: β-D-GlcpA-(1 → 3)-α-d-Galp A-(1 → 2)-l-Rha), JIM8 (sp: AGP), LM2 (AGP with ß-glucuronic acid), and LM6 (sp: (1 → 5)-alpha-arabinan/AGP epitopes). Primary antibodies were diluted 1/10 with growth medium before labeling of the algae. The secondary antibody used was goat-anti-rat TRITC (Sigma Chem. St. Louis, MO, United States) diluted 1/75 with growth medium. Control labeling was performed without primary antibody application. For *Spirogyra* rhizoids, coverslips containing rhizoids (above) were labeled by placing drops of antibody and washes onto the surface of the coverslips for the times indicated above.

For quantification of fluorescence signal of JIM13, we use three independent biological replicates to measure the fluorescence intensity using the transect function of the Olympus Fluoview 300 CLSM software. The fluorescence signal was estimated using six random segments in each biological sample. Equal threshold level was applied for each replicate to allow comparison among taxa. Data were analyzed with ANOVA in JMP version 12.0. Differences in the mean of fluorescence intensity signals were statistically tested using Tukey’s test (significance *P* < 0.05).

### Light and Confocal Laser Scanning Microscopy

For WFLM, samples were viewed with an IX-83 LM (Olympus) or BX 60 LM (Olympus). Samples for confocal laser scanning microscopy or CLSM were imaged with an Olympus Fluoview 300 CLSM. For TRITC imaging, we used the HeNe-G (Green/Red) laser, with 568nm excitation and 50% laser intensity. For samples treated with FITC and chlorophyll autofluorescence, we used the Ar laser with 488nm excitation and 10% laser intensity. For both, we used U-PLAN FL 20X/0.50 or UplanApo 60X/1.40 oil objectives. For samples analyzed with the CLSM, we chose the pseudo-colors for fluorescent signals that best high the labeling.

### Immunogold Labeling

Harvested cells were cryofixed, freeze substituted and processed for transmission electron microscope (TEM) using the methods described in Domozych et al. (2014). Spray freezing into liquid propane was employed for *Penium* and *Chlorokybus*. For multicellular taxa (*Spirogyra*, *Coleochaete*) samples were placed on 1 × 1 cm aluminum sheets, quickly blotted with filter paper to remove most growth medium and plunge frozen into liquid propane cooled with liquid nitrogen. *Zygnema* was frozen by a Leica EMPACT High pressure freezing (HPF) device and freeze substituted (Leica EM AFS) in 0.05% Ur-acetate and 0.1% OsO_4_ and embedded in London resin (LR) white according to Herburger and Holzinger (2015) and Holzinger and Pichrtová, 2016. For *Spirogyra* rhizoids, chopped filament fragments (see above) were allowed to settle onto sterile nitrocellulose sheets immersed in growth medium in a sterile petri dish. After rhizoid formation the sheets were placed on aluminum sheets as above and plunge frozen. 60 nm sections were cut with a Leica Ultramicrotome and collected on Formvar-coated nickel grids. Immunogold labeling followed the protocol of Domozych et al. (2017) or Herburger and Holzinger (2015) for *Zygnema*. Samples were imaged on a Zeiss Libra 120 TEM.

### ECM Isolation

For cell wall studies, cells were harvested by centrifugation 1,000 ×*g* for 1 min. The cell pellet was washed three times in fresh growth medium and re-centrifuged. Except for *Penium*, Alcohol Insoluble Residues (AIR) of cell walls were obtained using the technique of Foster et al. (2010). *Penium* cell walls were isolated via sonication/centrifugation method of Domozych et al. (2014). For EPS isolation, the growth medium of 10–14 day old cultures of *Penium*, *Chlorokybus* and *Zygnema* was collected by centrifugation at 3,000 ×*g* for 5 min to remove particulate materials. The supernatant was then recentrifuged at 10,000 ×*g* for 5 min and the supernatant was dialyzed against 4 l of distilled H_2_O (dialysis tubing cut off mw 3,000). The water was changed every 8 h for 2 consecutive days. The dialyzed supernatant was then collected and freeze dried.

### Protein Electrophoresis and Western Blotting

Freeze-dried samples were dissolved in deionized water (1 mg/mL) and mixed with 2× Laemmli Sample Buffer (Bio-Rad, Hercules, CA, United States) with 5% (v/v) β-mercaptoethanol. The samples were vortexed, boiled in a water bath for a total of 2 min, cooled on ice, and centrifuged at 12,000 rpm for 2 min. Twelve microliter aliquots of samples were loaded into wells of a Mini-Protean^®^ TGX^TM^ gel 4–20% (Bio-Rad, Hercules, CA, United States). Twelve microliter of the Precision Plus Protein unstained standards (Bio-Rad; cat #161-0363) were loaded as a control. Electrophoresis was performed in a Tris/Glycine/SDS buffer (Bio-Rad, Hercules, CA, United States) at 100 V, 1 A for 5 min, and then at 125 V, 1.25 A for 55 min.

The separated proteins were transferred onto a nitrocellulose membrane (NitroBind 0.45 μm, GVS North America) by electroblotting using a Trans-Blot^®^ SD Semi-dry Electrophoretic Transfer Cell (Bio-Rad, Hercules, CA, United States) at 12 V, 0.3 A, 2 h in Towbin transfer buffer (25 mM Tris, 192 mM glycine, 20 % (v/v) methanol, pH 8.3). The gel was placed in a box, and with constant gentle shaking, washed with water 3 × for 5 min, developed with Bio-Safe^TM^ Coomassie G-250 stain (Bio-Rad, Hercules, CA, United States) for 1 h, washed with water 3 × for 5 min, and de-stained overnight in water, until photographed.

The membrane was transferred to a plastic box. The Western blot steps were performed at RT, with constant gentle shaking. The membrane was incubated with a blocking solution containing 0.5% (w/v) blotting grade blocker (Bio-Rad) in PBST for 30 min, washed with PBST 3× for 5 min, and incubated overnight with 1:10 solution of JIM13 mAb with PBST. After a 5 min wash for 3× with PBST, the membrane was blocked, rewashed, and incubated for 90 min with a 1:50 solution of goat anti-rat IgG-peroxidase antibody (Sigma, St. Louis, MO, United States). After washed for 10 min 3× in PBS, the membrane was developed with a colorimetric detection Amplified Opti-4CN^TM^ Detection Kit (Sigma, St. Louis, MO, United States) and photographed.

## Results and Discussion

### Identification of Adhesion Sites Using Fluorescent Beads

**FIGURE 1:**
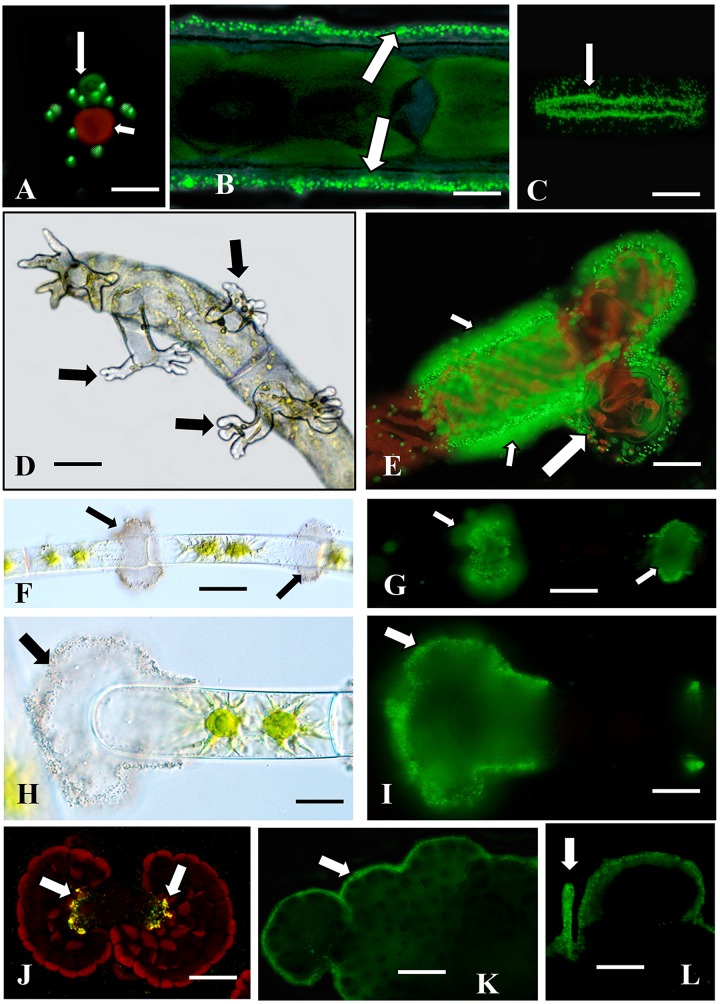
*Adhesion zones identified with fluorescent beads.* **(A)** *Chlorokybus* (small arrow) adheres to glass or plastic substrates. Fluorescent beads label the zone adjacent to the cell attachment site (large arrow). Bar 5.2 μm. **(B)** Bead labeling of the cell wall surface of *Penium* (arrows) after EPS was washed from the cell surface. Bar 45.3 μm. **(C)** Bead labeling of the adhesive “footprint” (arrow) that remains on the substrate after *Penium* is washed from substrate surface. Bar 45.3 μm. **(D)** Rhizoids (arrows) emerge from live cells immediately adjacent to the wound zone in a wounded *Spirogyra* filament. Bar 21.1 μm. **(E)** Bead labeling of a sheath that is external to the cell wall of growing rhizoids (large arrow). Note that the surface of the rhizoid producing cell also labels (small arrows). **(F,G)** Labeled “clouds” of beads are found at the cross-wall areas of planktonic *Zygnema* filaments (arrows). **(H,I)** Bead labeling at the sheath surrounding the cell at a fragmentations site (arrow). **(J)** Bead labeling of the center thallus zones of *Coleochaete* (arrows). **(K)** The beads also label the thallus margins (**K**, arrow) and the hairs emerging from the dorsal side of the thallus (**L**, arrow). Bars (**F–L**) 5.2 μm.

Many charophytes attach to various substrates through a wide variety of adhesion mechanisms. 0.5 μm Fluoresbrite beads were employed in simple and rapid labeling assays to identify adhesion zones on, or secreted from, our charophyte taxa. These beads adhere firmly to adhesive materials on the cell surface and can easily be imaged through WFLM or CLSM. *Chlorokybus* (Chlorokybales) exists either as a unicell or loosely organized in sarcinoid packets of 2–4 cells. While mainly planktonic in laboratory cultures, this alga will adhere to solid substrates (e.g., plastic, glass). Fluoresbrite bead-labeling of attached cells shows aggregations of beads on the substrate immediately adjacent to the cells (Figure 1A). This suggests an adhesive material or sheath secreted from the cell. Planktonic cells either weakly bind or do not bind to the beads. In the unicellular desmid, *Penium* (Zygnematales), the beads rapidly attach to the outer surface of the cell wall (Figure 1B). *Penium* also adheres firmly to the surface of solid substrates (e.g., glass or plastic) within seconds. When attached cells are gently dislodged from the substrate surface with a pipette-generated stream of fresh growth medium and the substrate is subsequently incubated in medium containing beads, a “footprint” of aggregated beads that matches the shape of the cell remain (Figure 1C). This demonstrates that the adhesive ECM material on the cell wall surface affixes rapidly and firmly to the substrate surface after cell-substrate interaction. If cells are allowed to sit on the substrate for longer than 5 min, the beads on the cell surface is displaced by the secretion of EPS (Supplementary Figure 1A). These observations demonstrate that initial adhesion of the cell to a substrate is due not to EPS but rather to adhesive materials on the outer cell wall surface.

*Spirogyra* (Zygnematales) forms mats of loosely aggregated unbranched filaments. However, when filaments are artificially wounded (i.e., chopped with a razor blade) and then returned to culture, filament segments sink, then physically contact the substrate surface and produce distinct rhizoids within 24 h (Figure 1D). Rhizoids firmly attach to the substrate and produce a sheath around the rhizoid branches that label with the beads (Figure 1E). The cells of filaments that are not wounded do not bind to the beads or only in weak fashion. However, in wounded filaments, the cells producing rhizoids and those immediately adjacent to the wound site, produce a sheath on the cell wall surfaces that label with the beads. These observations indicate that *Spirogyra* exhibit an adhesion mechanism that is turned on by wounding and manifests in the production of both highly branched rhizoids and an associated adhesive material.

*Zygnema* (Zygnematales) forms dense aggregates of unbranched filaments that closely adhere to each other and affix weakly to solid substrates. When filaments are incubated with beads, distinctive “clouds” of adhesive material are visible at cross wall regions between cells of filaments (Figure 1F,G). Also, filaments fragment in culture. At the fragmentation site, the cell wall of the cell that will ultimately dislodge (i.e., terminal cell) swells (Figure 1H). Fluoresbrite beads distinctly label a sheath at this zone (Figure 1I). These observations suggest that the adhesive clouds found along the filament surfaces may be responsible for the filaments forming dense aggregates. This growth habit would be advantageous for the alga by serving as a means for water retention during desiccation stress in ephemeral wetland habitats or for maintaining close contact of the gametic cells of filaments for conjugation. The adhesive sheath that coats the swollen cell wall of the terminal cell at a fragmentation site may function like the sheath surrounding *Spirogyra* rhizoids, i.e., for rapid attachment to a substrate.

*Coleochaete orbicularis* (Coleochaetales) forms a pseudo-parenchymatous thallus that adheres firmly to solid substrates. When incubated with beads, labeling appears at three zones; (a) a narrow zone on the top surface of the thallus (Figure 1J), (b) along the cell peripheries of the outer edge of the thallus (Figure 1K), and (c) along the hairs emerging from the dorsal side of the thallus (Figure 1L). These multiple sites of adhesive materials most likely are responsible for this alga firm adhesion to substrates.

In order to elucidate the biochemical components of the adhesive materials, we labeled the charophytes with specific monoclonal antibodies raised against AGP epitopes (e.g., JIM13, LM6, JIM8, and LM2) and pectin (JIM5). Table 1 provides a summary of our results and specific details concerning labeling of specific taxa. In general, we found that all Charophytes positive bind to JIM13, JIM8, and JIM5. The remaining labeling show specificity to the different taxa (Table 1). We quantified the fluorescence intensity signal of AGP labeled by JIM13 and found that *Spirogyra* and *Zygnema* delimit the lower and upper range of fluorescence intensity of the charophytes, showing significant differences among them (Supplementary Figure 3).

**Table 1:** Immunofluorescence labeling of the ECM.

Taxon	JIM5	LM6	JIM13	JIM8	LM2	Yariv
*Chlorokybus*	+	+	+	+	⊖	⊖
*Coleochaete*	+	+	+	+	⊖	⊖
*Penium*	+	+	+	+	+	⊖
*Zygnema*	+	+	+	+	⊖	+
*Spirogyra*	+	⊖	+	+	+	⊖
*Spirogyra (Rizhoids)*	+	⊖	+	+	+	+


*(+) indicates positive binding. (⊖) indicates negative binding.*

#### *Chlorokybus* and an AGP Sheath for Adhesion

**FIGURE 2:**
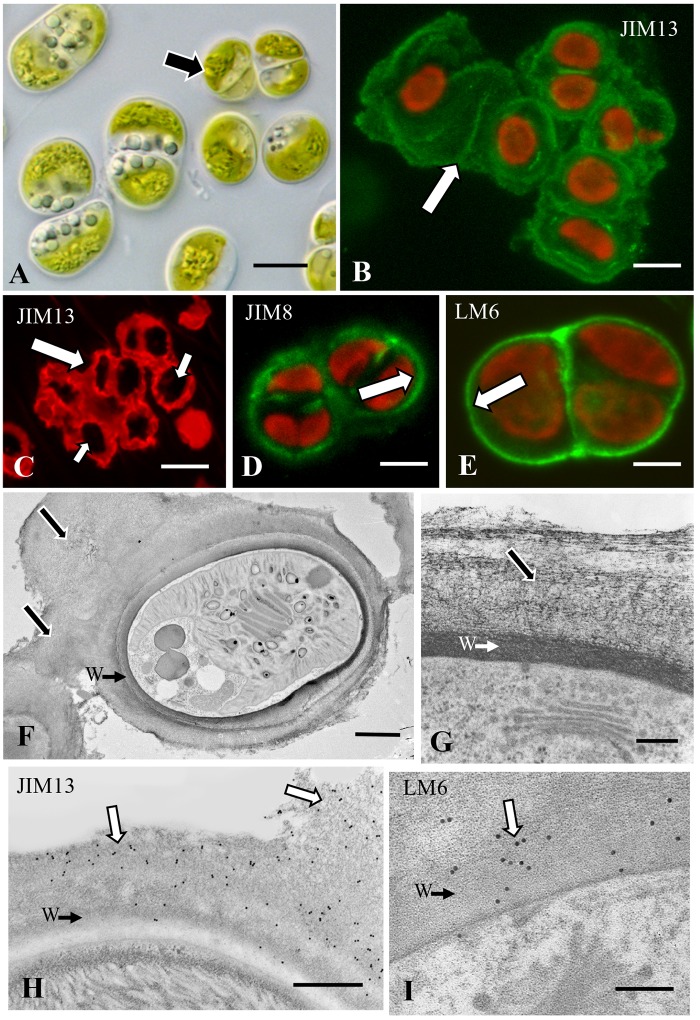
*Chlorokybus*: **(A)** DIC image of *Chlorokybu*s showing unicellular or sarcinoid packet form (arrow). Bar 6.5 μm. **(B)** JIM13 immunofluorescence demonstrating labeling in an extracellular sheath (arrow). Bar 6.0 μm. **(C)** JIM13 immunofluorescence CLSM image showing the extensive extracellular sheath both around and between cells (large arrow). The cells are the dark zones within the packet sheath (small arrows). Bar 9.0 μm. **(D)** JIM8 labeling of the cell wall (arrow). Bar 7.0 μm. **(E)** LM6 labeling of the cell wall (arrow). Bar 4.5 μm. **(F)** TEM image of a cell in a sarcinoid packet. Note the cell wall (CW→) surrounded by the extensive extracellular sheath (arrows). Bar 4 μm. **(G)** Magnified TEM image of the ECM showing the cell wall (CW→) and extracellular sheath (arrow). Bar 500 nm. **(H)** JIM13 immunogold labeling of the extracellular matrix (ECM, arrows), labeling of the cell wall very limited (CW→). Bar 700 nm. **(I)** LM6 labeling of the cell wall (arrow) and interface of the wall (W→) with the ECM. Bar 800 nm.

Single celled and packet-forming *Chlorokybus* (Figure 2A) produce an external sheath that labels with the mAb, JIM13 (Figure 2B,C). This antibody recognizes β-D-GlcpA-(1 → 3)-α-d-Galp A-(1 → 2)-l-Rha that is part of the glycan portion of AGPs. This labeled sheath is extensive (e.g., up to 2–4× the diameter of a cell) and is positioned both between and surrounding the cells/packets cells (Figure 2B,C). Two other AGP-binding mAbs, JIM8 and LM6 (Lee et al., 2006; Huang et al., 2013), also label the cell surface but labeling is restricted to the cell walls and not the external sheaths (Figure 2D,E). The external sheath and cell walls of *Chlorokybus* do not label with ß-glu-Yariv nor did the Yariv reagent restrict labeling with JIM13. TEM imaging of *Chlorokybus* highlights the extensive sheath found external to the cell wall (Figure 2F). Closer examination shows that this sheath is an aggregate of loose fibrillar constituents that sit on a cell wall made of highly dense fibrils (Figure 2G). Immunogold labeling with JIM13 labels the wall/sheath ECM but is more apparent on the sheath (Figure 2H). LM6 labeling (Figure 2I) was more prevalent on the cell wall as was JIM8 labeling (results not shown). We also examined both the culture supernatant and AIR-processed cell walls via electrophoretic separation and Western blotting with JIM13 (Supplementary Figure 2). For cell walls, a high molecular weight band and a broad band or “smear” ranging from 20 to 60 kD are noted. These results show that AGP is located throughout the ECM and is ultimately released into the culture medium.

The results presented here suggest that AGPs constitute CAMs in an early divergent charophyte, *Chlorokybus*. These CAMs are most likely responsible for the formation of loose sarcinoid packets as well as for attachment to solid substrates. Future detailed biochemical analyses will be needed in order to provide critical information that can be used for comparative studies with the large assortment of proteoglycans that constitute the AGP.

#### *Spirogyra*, AGP and Wound-Stimulated Rhizoids

**FIGURE 3:**
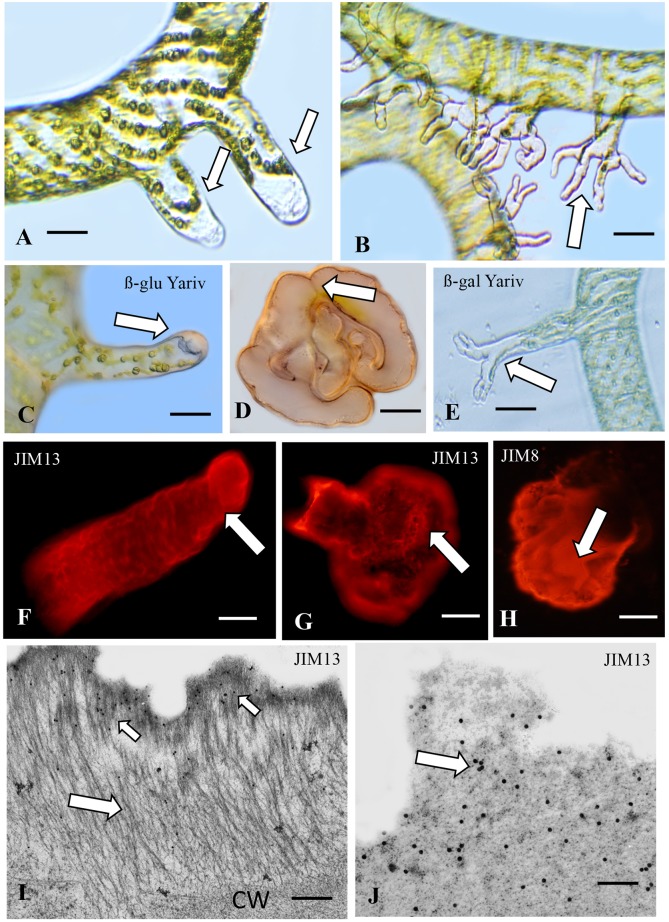
*Spirogyr*a. **(A)** DIC image of young (24 h old) unbranched rhizoids (arrows) emerging from a wounded filament. Bar 48 μm. **(B)** DIC image of mature (36 h old) branched rhizoids (arrow). Bar 65 μm. **(C)** ß-glucosyl-Yariv labeling of the polar tip (arrow) of a young unbranched rhizoid. Bar 20 μm. **(D)** Yariv reagent labeling of a mature branched rhizoid (arrow) at its attachment point on a glass substrate. Bar 50 μm. **(E)** Yariv control. No labeling (arrow) with β-galactosyl Yariv. Bar 25 μm. **(F)** JIM13 labeling of a sheath on the outer surface of a young unbranched rhizoid (arrow). **(G)** JIM13 labeling of the sheath surrounding mature branched rhizoids (arrow). Bar 40 μm. **(H)** JIM8 labeling of the sheath surrounding mature branched rhizoids (arrow). Bar 50 μm. **(I)** TEM image of the ECM of a young rhizoid. External to the cell wall (CW) emerges a fibrillar sheath (large arrow). Limited JIM13 labeling is apparent on the sheath surface (small arrows). **(J)** JIM13 labeling of the sheath of mature rhizoids (arrow). Bars (**I,J**) 600 nm.

Twenty four hours after wounding *Spirogyra* filaments, short unbranched rhizoids emerge from the cell or cells closest to the wound site (Figure 3A) and use the rhizoids for attachment to surrounding substrates. Quickly thereafter, extensive polar expansion-based branching of these rhizoids yields finger-like rhizoids that firmly attach to a substrate (Figure 3B). The cell wall at the expanding tip of the young unbranched rhizoids label with ß-glu-Yariv reagent (Figure 3C) as do the cell wall and sheath surrounding the mature branched rhizoids (Figure 3D). When freshly wounded filament fragments are placed in growth medium containing 5, 10, or 20 μM Yariv reagent, rhizoid formation is inhibited. Control labeling with alpha-gal-Yariv does not result in any labeling of any stage of rhizoid formation (Supplementary Figure 1B). Also, control *Spirogyra* filaments (i.e., not wounded) do not label with Yariv reagent (Figure 3E). These results represent the first time that ß-glu-Yariv reagent has been shown to label the ECM of charophytes. ß-glu-Yariv reagent is a synthetic phenylglycoside that is a standard screening agent for identifying, quantifying and purifying classical AGPs and glycoproteins with AGP domains in higher plants (Mashiguchi et al., 2008). JIM13 labels a sheath coating the outer surface of the young, unbranched rhizoids (Figure 3F) and the extensive sheath surrounding the mature branched rhizoids (Figure 3G). JIM8 (Figure 3H) and LM6 also label the sheath (Supplementary Figure 1D). Western blotting with JIM13 labeled multiple bands in cell walls and several bands at high molecular weight in the rhizoids (>75 kD; Supplementary Figure 2). Labeling with the mAb, JIM5 demonstrates that homogalacturonan is also part of the rhizoid cell wall (Supplementary Figure 1E). TEM analysis shows that the sheath of young rhizoids consists of aggregates of fibrils (Figure 3I) on the surface of the cell wall that label with JIM13 (Figure 3I). This labeling is more abundant in the sheath of mature branched rhizoids (Figure 3J).

When wounded, *Spirogyra* produces rhizoids for attachment to substrates. Rhizoid formation in *Spirogyra* has been studied in the past and has been shown to require distinct environmental cues and reactive signaling mechanisms (Nagata, 1979; Inoue et al., 2002; Kim et al., 2005; Ikegaya et al., 2008). In this alga, the production of a cellular structure (rhizoid) with a large surface area (branched) and coated with a highly adherent-CAM that contains AGP would be critical for rapid attachment to a substrate upon wounding. Interestingly, the production of rhizoids/CAM occurs in cells only near the wound site. This suggests a finely tuned sensing mechanism that ultimately leads to the formation of rhizoids/CAM from specific cells at the precise wound zone. This morphogenetic specialization that would require activation of multiple morphogenesis-directing gene sets at specific thallus sites is a common characteristic exhibited in all plant groups (Jones and Dolan, 2012; Proust et al., 2016). The influence of ethylene as a phytohormone responsible for cell wall modifications in *Spirogyra* was illustrated by Van De Poel et al. (2016). Finally, the presence of pectin in the rhizoid cell walls suggests the possibility of interacting pectic-AGP polymers similar to that observed in land plants (Huang et al., 2016).

#### *Zygnema* and Fragmentation

**FIGURE 4:**
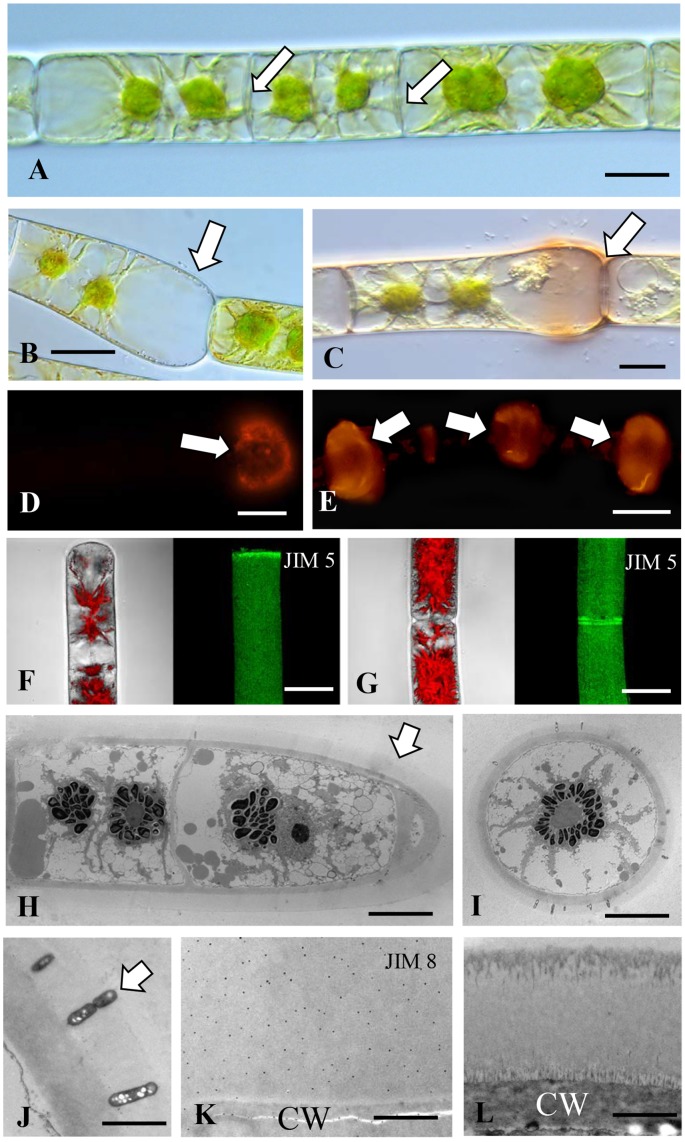
*Zygnema*: **(A)** Cross walls of the unbranched filament (arrows). Bar7 μm. **(B)** Cell wall (arrow) at swelling of cell at a fragmentation site (arrow). Bar 12 μm. **(C)** ß-glucosyl-Yariv labels the wall and sheath of the swollen zone (arrow). Bar 12 μm. **(D)** JIM13 labeling of ECM at the swollen fragmentation zone (arrow). Bar 15 μm. **(E)** JIM13 labeling of “clouds” of ECM at the cross walls of filaments (arrows). Bar 15 μm. **(F)** JIM5 labeling (right) of terminal cell and corresponding bright field image with chloroplast autofluoescence (left). **(G)** JIM5 labeling of two connected cells, notice the two rings in the connection zone (arrow). Bars (**F**,**G**) 20 μm. **(H)** TEM image of young filament with broad electron translucent ECM layer surrounding the cells (arrow). **(I)** Cross section through filament. Bars (**H**,**I**) 20 μm. **(J)** Bacteria in the ECM layer of a young filament (arrow). **(K)** JIM8 immuno-gold labeling of the ECM layer outside the cell wall (CW). **(L)** ECM of older cell, notice the fibrillary structures outside the CW and at the periphery. Bars (**J**–**L**) 2 μm.

Our work with *Spirogyra* led us to examine other Zygnematalean filamentous taxa for the ability to produce rhizoids and AGP-containing CAMs. Like *Spirogyra*, *Zygnema* produces unbranched filaments (Figure 4A) but rhizoids are not induced by the wounding protocol used for *Spirogyra*. In *Zygnema* rhizoids have never described before (e.g., Herburger et al., 2015). *Zygnema* filaments though often fragment in laboratory culture. As mentioned previously, the fragmentation site is marked by a swollen cell wall of the terminal cell at the fragmentation site (Figure 4B). This effect might be due to turgor induced changes and rounding of the terminal cell has frequently been observed (Kaplan et al., 2013). This wall zone and surrounding sheath labels with Yariv reagent (Figure 4C) and with JIM13 (Figure 4D). JIM8 provides a similar labeling profile but LM6 does not label the sheath but weakly labels the cell walls of all *Zygnema* cells (Supplementary Figures 1F,G). Unlike, the cross walls of most cells of the filament also produce a sheath that labels with JIM13 (Figure 4E). JIM5 stains the whole surface of the cell wall, the strongest signal is on the tips or where two cells are connected (Figure 4F,G). TEM imaging illustrates that outside of the cell wall a 2–3 μm broad electron translucent ECM is present (Figure 4H,I). This ECM may contain bacteria (Figure 4I,J), when stained with JIM8 this outer ECM layer is stained (Figure 4K). In older cells, this layer becomes more electron dense and shows a fibrillary structure close to the cell wall and toward the periphery (Figure 4L). Western labeling of AIR-processed *Zygnema* cell walls and the culture supernatant reveal labeling of high molecular weight bands (Supplementary Figure 2). We assume that the AGP remained during cell wall processing as we were unable to obtain *Zygnema* cells from just fragmentation areas. The functional role of AGP at cross wall sites on the filaments will require further investigation but they may allow for holding filaments together in aggregates or for assuring a CAM is readily available at any potential fragmentation site. While the exact mechanism for fragmentation positioning is not resolved, this process entails a swelling of the cell wall of the cell at the fragmentation site. This zone also contains AGP-containing CAM. Additionally, JIM5 labeling of its cell walls suggests putative interaction between AGP and pectin in adhesion zones. The distribution of pectins (in this case the partially methyl-esterified homogalacturonans) and their role in water holding in *Zygnema* is part of a study by Herburger et al. (2019) to the Frontiers research topic: *Co-Evolution of Plant Cell Wall Polymers*. Particularly in young cells JIM5 labeling occurs at the tips of fragmented cells. For this antibody an age dependent pattern in the outer cell walls has been found as described (Herburger et al., 2019).

#### *Penium margaritaceum* the “Super” Sticker

**FIGURE 5:**
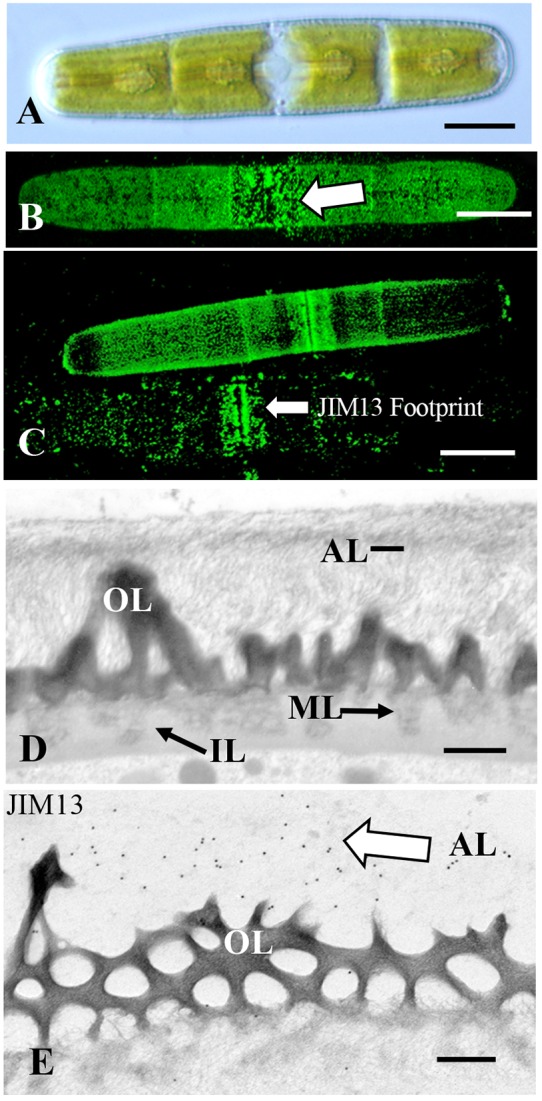
*Penium*: **(A)** DIC image of *Penium*. Bar 12 μm. **(B)** JIM13 labeling of the outer surface of the cell wall (arrow). Bar 15 μm. **(C)** JIM13 footprint (arrow) of adhesive material left on substrate after removal of cells. Bars 15 μm. **(D)** TEM image of the cell wall revealing the pectin-rich outer wall (OL) that connects to a medial layer (ML) that is embedded in an inner layer (IL). External to the outer layer is an adhesive layer (AL) of fine fibrils (arrows). Bar 300 nm. **(E)** JIM13 labeling (arrow) of the outer adhesive layer (AL) of the wall. Bar 250 nm.

*Penium* is a cylindrical unicellular desmid (Figure 5A) that readily adheres to multiple substrates. Previous work has shown that an extensive EPS is secreted beyond its cell wall that both fuels gliding motility and also contributes to the cell sticking to a substrate (Domozych et al., 2005). In this study, we show that if the EPS is removed by extensive washing, *Penium* still adheres rapidly and firmly to various substrates (Figure 1B). JIM13 labeling reveals the presence of AGP epitopes on the outer surface of the cell wall (Figure 5B) and in the aforementioned “footprints” (Figure 5C), i.e., cell surface material that is left on the substrate when cells are removed. JIM8 and LM6 also label the outer surface of the cell wall but less intensely than JIM13 (Supplementary Figures 1I,J). JIM5 labels the outer layer homogalacturonan in the cell wall (Supplementary Figure 1K). However, Yariv reagent does not label the cell wall, footprint or EPS of *Penium*. TEM analysis of the cell wall reveals a dense aggregate of fine fibrils emerging from the surface of the cell wall (Figure 5D) that labels with JIM13 (Figure 5E). Western screening with JIM13 reveals broad bands (Supplementary Figure 2). These results show that AGP found on the outer surface of the cell wall contributes to cells rapidly and firmly attaching to a substrate. Unlike *Chlorokybus*, *Spirogyra*, and *Zygnema*, the AGP is found all over the cell surface. This is most likely critical for efficient attachment when cells dislodged from sites transition back to the sessile habit. EPS secretion after attachment that fuels gliding and ensheathment on a surface also contains AGP which may assist in latter attachment of cells to a substrate. Finally, the distinct pectin lattice of the *Penium* cell wall (Domozych et al., 2014) is in close contact with the adhesive fibrils that once again indicates AGP-pectin interactions.

*Penium margaritaceum* has been considered as the most distant outgroup in the phylogenetic tree of Desmidiaceae by Gontcharov and Melkonian (2011). The various groups of desmids might have a different ecology, and the sticking abilities of *Penium* are quite different than that of other “saccoderm” desmids like *Netrium digitus* (Eder and Lütz-Meindl, 2010) or “placoderm” desmids like *Micrastieras* (Brosch-Salomon et al., 1998; Lütz-Meindl and Brosch-Salomon, 2000). In the latter, AGPs have been detected during the formation of primary cell walls, but as the cell walls mature (formation of secondary cell walls) their occurrence has been detected exclusively along the plasma membrane of the non-growing semicell indicating a regulatory role in growth (for a summary see Lütz-Meindl, 2016).

#### Coleochaete

**FIGURE 6:**
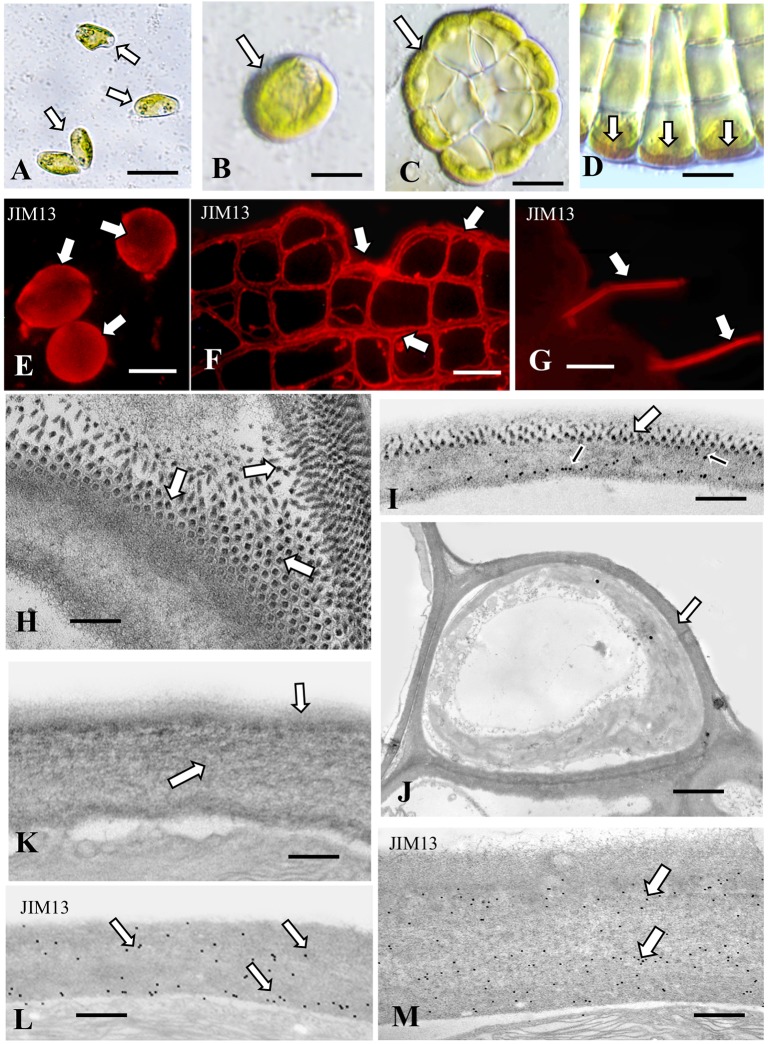
*Coleochaete*: **(A)** Zoospores (arrows) that have adhered to the substrate. Bar 5 μm. **(B)** Vegetative cell (arrow) after zoospore settling and cell wall production. Bar 8 μm. **(C)** 96 h old vegetative multicellular thallus (arrow). Bar 12 μm. **(D)** Yariv reagent labeling of cell walls (arrows) at the periphery of the vegetative thallus. Bar 10 μm. **(E)** JIM13 labeling of zoospore cell walls (arrows). Bar 8 μm. **(F)** JIM13 labeling of the vegetative thallus (arrows). Bar 10 μm. **(G)** JIM13 labeling of the hairs (arrows) that emerge from the thallus. Bar 10 μm. **(H)** TEM view of the zoospore cell wall surface. Note the ordered packing of projections (arrows). Bar 300 nm. **(I)** JIM13 labeling of the zoospore cell wall. Labeling is found throughout the wall (arrows). Large arrows show the projection found in the outside of the cell wall of young *Coleochaete*. Small arrows represent JIM13. Bar 1 μm. **(J)** TEM image of an outer vegetative cell. Note the thick cell wall (arrow). Bar 2.25 μm. **(K)** TEM image of the vegetative cell wall. An inner wall layer of tightly packed fibers (large arrow) subtends a thin layer of fibrils (small arrow). Bar 450 nm. **(L)** JIM13 labeling of the cell wall of a vegetative cell. Note the labeling through the wall (arrows). Bar 500 nm. **(M)** JIM13 labeling of the hair cell wall. Bar 300 nm.

*Coleochaete orbicularis* forms a discoidal, single layered, pseudoparenchymatous thallus that adheres firmly to multiple substrates. Upon subculture, zoospores are often released from undifferentiated cells of the thallus. The zoospores eventually settle on and attach to the underlying substrate (Figure 6A). Shortly thereafter the zoospore expands, produces a thick cell wall (Figure 6B) and subsequently undergoes cell division to produce the discoidal thallus (Figure 6C). The Yariv reagent labels an inner layer of the cell wall of the outermost cells of the thallus (Figure 6D). The thallus but not the hairs also label with JIM8 (Supplementary Figure 1L) and no part of the thallus labels with JIM8. JIM5 labels cell walls throughout the thallus (Supplementary Figure 1M). Western analysis of the cell walls shows labeling of band at a molecular weight of 150 kD (Supplementary Figure 2). The extensive presence of an AGP-like CAM most likely contributes to the firm attachment of thalli and zoospores to the surface of substrates. TEM analysis reveals that the cell wall of a settled adherent zoospore consists of an inner fibrous layer with an attached layer of projections that tightly pack on the outer surface (Figure 6H). JIM13 labeled all parts of this wall (Figure 6I). The role of the wall projections is not known and requires further investigation. When the multicellular thallus forms, each cell is covered by a cell wall (Figure 6J) that consists of a densely fibrous inner layer that subtends a thin outer layer of less dense fibrils (Figure 6K). The cell wall of a hair consists of a thick layer of packed fibers (Figure 6L). Both the hair cell wall and vegetative cell wall label with JIM13 (Figure 6L,M). These results show that AGP is a component of the cell walls of all cell types of *Coleochaete*. This is supported by labeling with JIM8 and LM6, mAbs that also recognize AGP epitopes. This may contribute to its firm adhering properties. In a recent transcriptomic study, *Coleochaete* was found to up- and down-regulate various cell wall-associated enzymes under stress and stood out in terms of its suite of potential cell wall modification proteins (De Vries et al., 2017, 2018). The potential for desiccation tolerance in *Coleochaete* being one of the triggers for initial terrestrialization was illustrated by Graham et al. (2011). Control labeling of all five organisms whereby primary antibodies were eliminated during the labeling protocol reveals no wall or sheath labeling (Supplementary Figures 1N–R).

## Conclusion

Adhesion is a common phenomenon exhibited by charophytes. To be able to stick on a surface is critical for many functions ranging from holding cells together in a multicellular thallus and more often for attaching to substrates. A common attribute of most early divergent organisms is adhesion to a substrate as they establish and maintain their existence in a biofilm, i.e., an interactive community of diverse taxa that enhances growth and development. Fluorescence quantification of JIM13 labeling yield significant differences between two filamentous members of Zygnematophyceae; *Spiorogyra* with the lowest signal, while *Zygnema* showed the highest signal. These results can possibly be interpreted by the ecological background where *Zygnema* forming extensive mats (e.g., Holzinger et al., 2009) might benefit from sticking the filaments together, a feature not necessary in the strictly aquatic *Spiorgyra*. From the other examined algae, *Chlorokybus*, *Penium*, and *Coleochaete*, only the latter was significantly different from *Zygnema* in the fluorescence intensity.

For extant and ancient charophytes, a rapid and effective adhesion mechanism would be especially critical in highly changeable wetlands. Additionally, adhesion of the thallus to a stable position on a substrate would enhance light absorption for photosynthesis and also create a direct physical conduit for capillary water movement from the substrate that would combat desiccation. Recent work has shown that charophytes live in complex biofilm communities (Domozych and Domozych, 2008) and a rapid adhesion mechanism would be critical for establishment of these communities. Further work will be required to elucidate the biology of extant charophyte-associated biofilms that would also provide insight into the role of these communities in the invasion of ancient terrestrial habitats.

This study demonstrates that the adhesion mechanism in various charophyte taxa is a result of the positioning of CAMs in the ECM at specific sites of the thallus or cell. This includes the sheath surrounding rhizoids or the thallus and the outer layer of the cell wall. After surveying a diverse taxonomic assemblage of charophytes, we provide evidence that indicates that AGPs constitute one part of the components that are secreted and used in adhesion phenomena. Thus, in this study, we suggest that AGPs participate in adhesion. AGPs are just one group of the large assortment of hyp-containing proteoglycans that have been found in all plant groups (Lee et al., 2006; Huang et al., 2013) and even other eukaryotes (Herve et al., 2016). The presence of AGPs in the taxa studied here and in the wall pores of the desmid, *Pleurotaenium* (Domozych et al., 2007) show that AGPs are employed at very different geographic zones and during different developmental stages of charophytes. It might be also surmised then that AGPs also evolved to play key roles in adhesion and other functions in land plants. Further work into the biochemistry and molecular biology of charophyte AGPs will be necessary for understanding the functional and evolutionary significance of these proteoglycans.

## Author Contributions

DD and AH designed research. KP-L and BT performed research. KP-L, AH, and DD wrote the manuscript.

## Conflict of Interest Statement

The authors declare that the research was conducted in the absence of any commercial or financial relationships that could be construed as a potential conflict of interest.

## Footnotes

1 https://utex.org/products/modified-bolds-3n-medium

2 http://www.plantprobes.net/index.php
