# Supplementary material for: Arabinogalactan Proteins and the Extracellular Matrix of Charophytes: A Sticky Business
Source: Front Plant Sci. 2019 Apr 12;10:447. doi: 10.3389/fpls.2019.00447 (PMC6474363; doi:10.3389/fpls.2019.00447)
Supplement: Supplementary file 1 [file Presentation_1.pptx]

## Slide 1
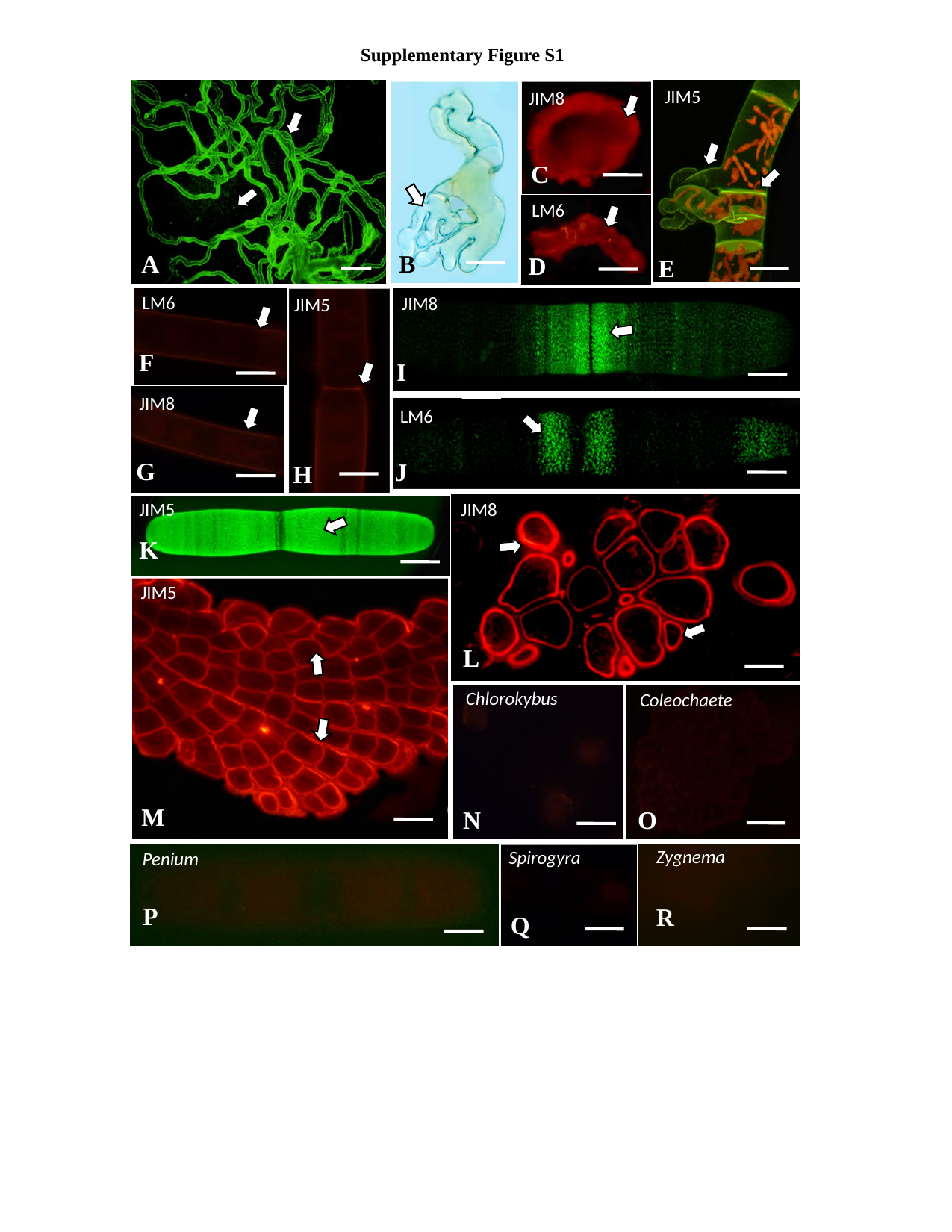

Supplementary Figure S1
JIM5
JIM8
C
LM6
A
B
D
E
LM6
JIM8
JIM5
F
I
JIM8
LM6
G
J
H
JIM5
JIM8
K
JIM5
L
Chlorokybus
Coleochaete
M
O
N
Zygnema
Spirogyra
Penium
P
R
Q
R

## Slide 2
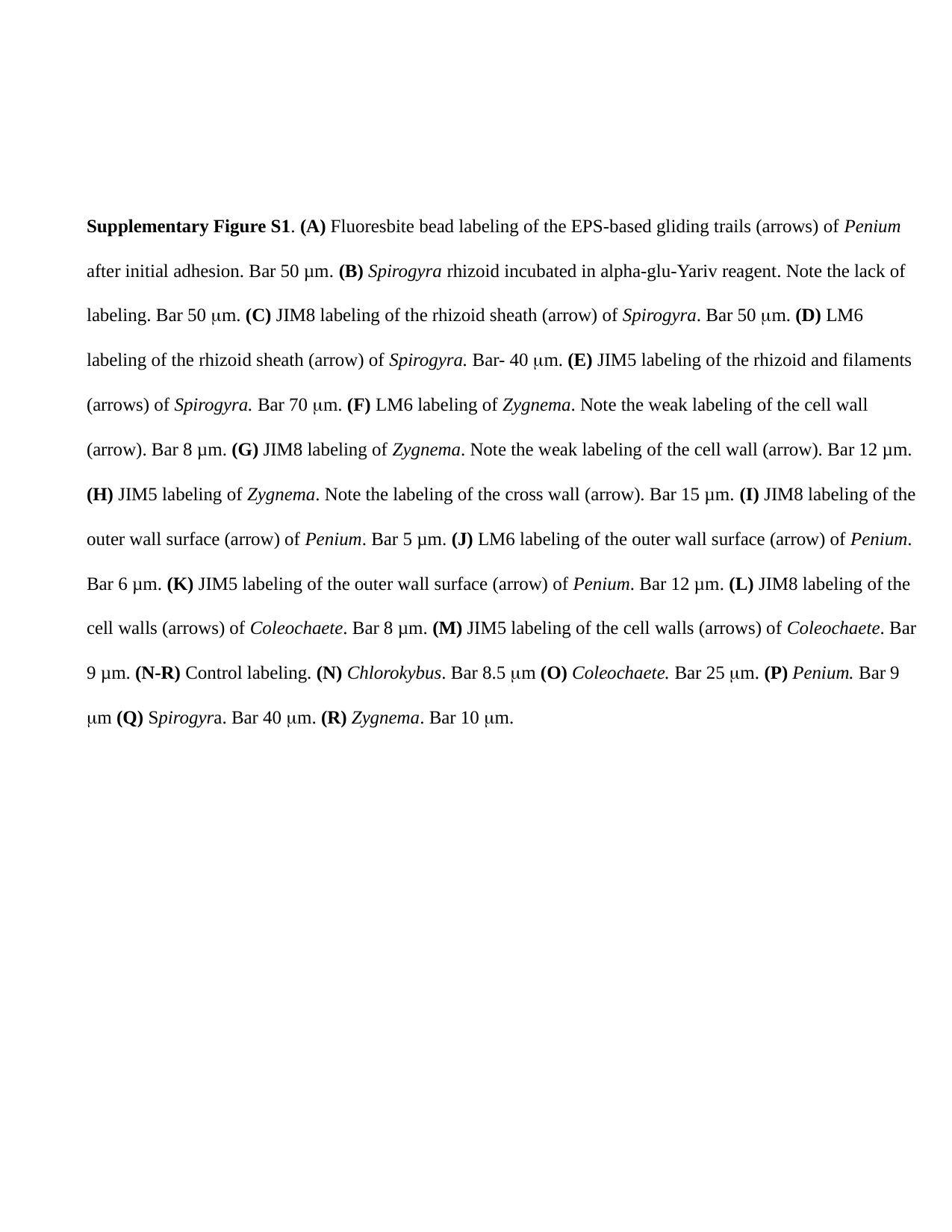

Supplementary Figure S1. (A) Fluoresbite bead labeling of the EPS-based gliding trails (arrows) of Penium after initial adhesion. Bar 50 µm. (B) Spirogyra rhizoid incubated in alpha-glu-Yariv reagent. Note the lack of labeling. Bar 50 μm. (C) JIM8 labeling of the rhizoid sheath (arrow) of Spirogyra. Bar 50 μm. (D) LM6 labeling of the rhizoid sheath (arrow) of Spirogyra. Bar- 40 μm. (E) JIM5 labeling of the rhizoid and filaments (arrows) of Spirogyra. Bar 70 μm. (F) LM6 labeling of Zygnema. Note the weak labeling of the cell wall (arrow). Bar 8 µm. (G) JIM8 labeling of Zygnema. Note the weak labeling of the cell wall (arrow). Bar 12 µm. (H) JIM5 labeling of Zygnema. Note the labeling of the cross wall (arrow). Bar 15 µm. (I) JIM8 labeling of the outer wall surface (arrow) of Penium. Bar 5 µm. (J) LM6 labeling of the outer wall surface (arrow) of Penium. Bar 6 µm. (K) JIM5 labeling of the outer wall surface (arrow) of Penium. Bar 12 µm. (L) JIM8 labeling of the cell walls (arrows) of Coleochaete. Bar 8 µm. (M) JIM5 labeling of the cell walls (arrows) of Coleochaete. Bar 9 µm. (N-R) Control labeling. (N) Chlorokybus. Bar 8.5 μm (O) Coleochaete. Bar 25 μm. (P) Penium. Bar 9 μm (Q) Spirogyra. Bar 40 μm. (R) Zygnema. Bar 10 μm.

## Slide 3
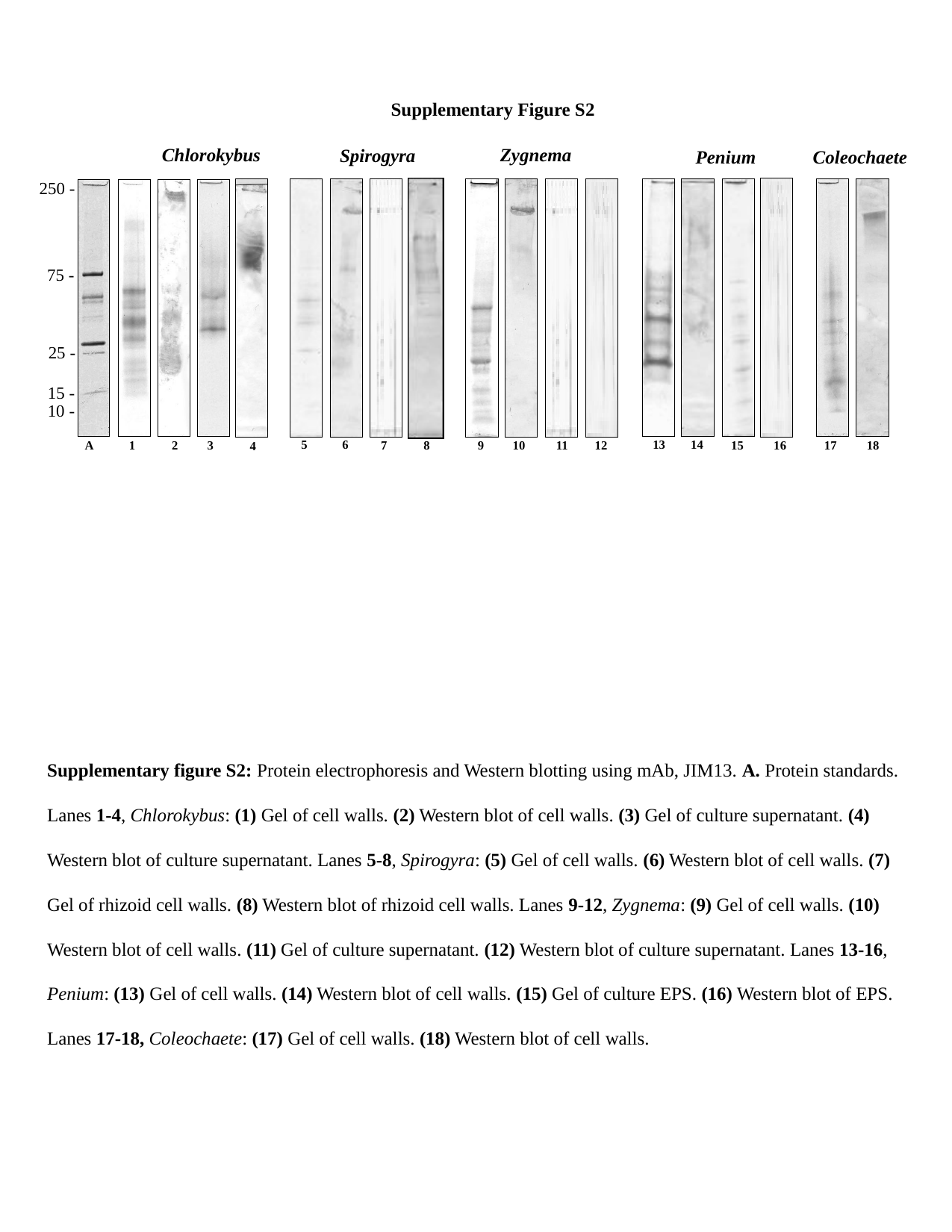

Supplementary Figure S2
Chlorokybus
Zygnema
Spirogyra
Coleochaete
Penium
250 -
75 -
25 -
15 -
10 -
13
14
5
6
1
2
17
18
15
16
A
9
10
7
8
11
12
3
4
Supplementary figure S2: Protein electrophoresis and Western blotting using mAb, JIM13. A. Protein standards. Lanes 1-4, Chlorokybus: (1) Gel of cell walls. (2) Western blot of cell walls. (3) Gel of culture supernatant. (4) Western blot of culture supernatant. Lanes 5-8, Spirogyra: (5) Gel of cell walls. (6) Western blot of cell walls. (7) Gel of rhizoid cell walls. (8) Western blot of rhizoid cell walls. Lanes 9-12, Zygnema: (9) Gel of cell walls. (10) Western blot of cell walls. (11) Gel of culture supernatant. (12) Western blot of culture supernatant. Lanes 13-16, Penium: (13) Gel of cell walls. (14) Western blot of cell walls. (15) Gel of culture EPS. (16) Western blot of EPS. Lanes 17-18, Coleochaete: (17) Gel of cell walls. (18) Western blot of cell walls.

## Slide 4
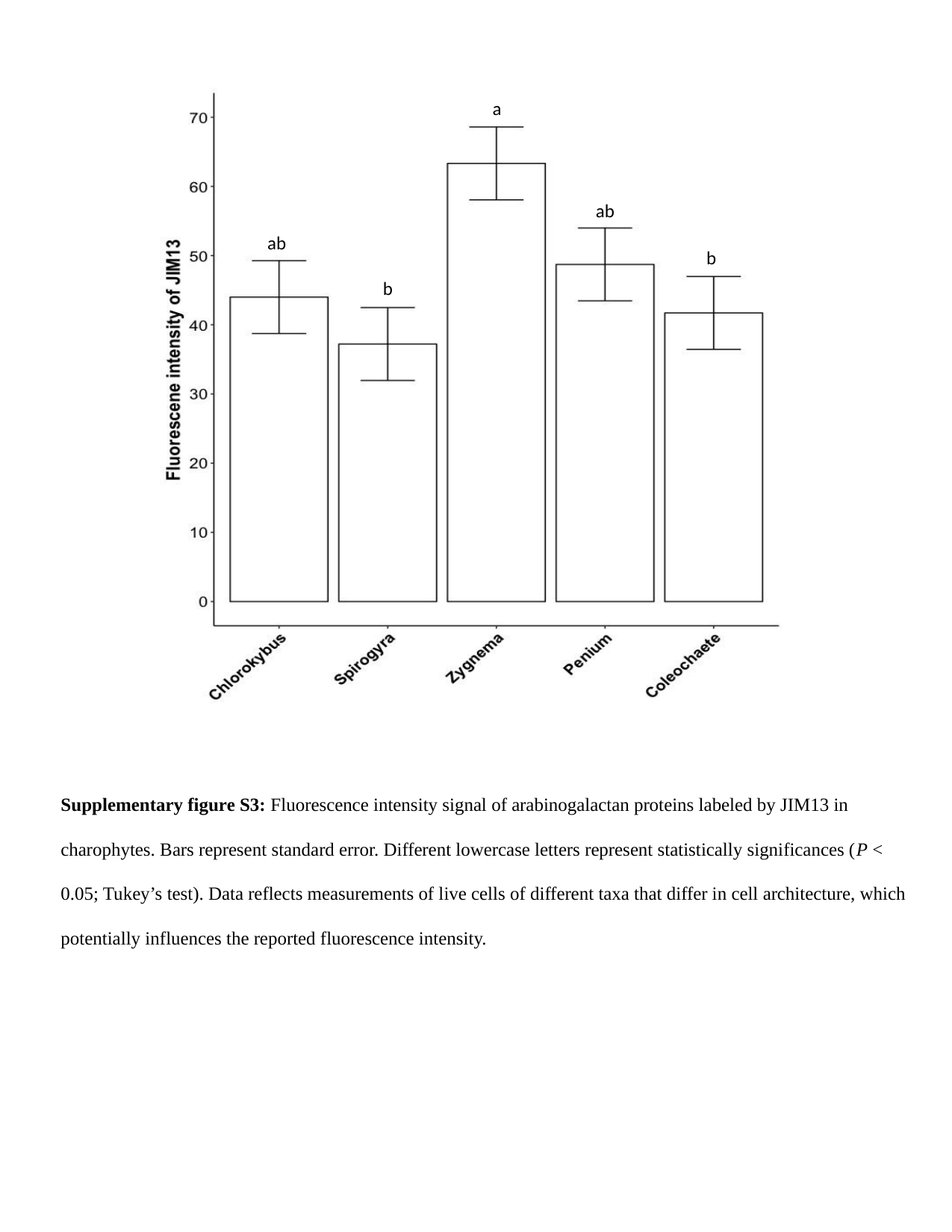

a
ab
ab
b
b
Supplementary figure S3: Fluorescence intensity signal of arabinogalactan proteins labeled by JIM13 in charophytes. Bars represent standard error. Different lowercase letters represent statistically significances (P < 0.05; Tukey’s test). Data reflects measurements of live cells of different taxa that differ in cell architecture, which potentially influences the reported fluorescence intensity.
